# Total Hip Arthroplasty Is Associated With Lower 90-Day Inpatient Readmission Than Hemiarthroplasty for Femoral Neck Fracture in Patients Aged 65-75 Years: A Propensity Score-Matched Nationwide Readmissions Database Study

**DOI:** 10.1016/j.artd.2026.102168

**Published:** 2026-10-06

**Authors:** David Maman, Yaniv Steinfeld, Yaron Berkovich

**Affiliations:** a The Ruth and Bruce Rappaport Faculty of Medicine, Technion Israel Institute of Technology, Haifa, Israel; b Department of Orthopedic Surgery, Carmel Medical Center, Haifa, Israel

**Keywords:** Femoral neck fracture, Total hip arthroplasty, Hemiarthroplasty, Readmission, Health economics

## Abstract

**Background:**

Comparative early hospital-based outcomes of hemiarthroplasty and total hip arthroplasty (THA) for femoral neck fracture remain uncertain in patients aged 65-75 years.

**Methods:**

Using the 2022 Nationwide Readmissions Database, we identified patients aged 65-75 years admitted with an acute femoral neck fracture who underwent hemiarthroplasty or THA. Patients were matched 1:1 using exact matching on age, Hospital Frailty Risk Score category, and mental-disorder status, plus propensity-score matching with a 0.20-standard-deviation caliper. The primary outcome was all-cause inpatient readmission within 90 days.

**Results:**

Among 8,012 eligible admissions, 5,108 patients underwent hemiarthroplasty and 2,904 underwent THA. Matching yielded 2,702 patients per group, with all postmatch absolute standardized mean differences <0.10. THA was associated with a lower observed 90-day inpatient readmission rate than hemiarthroplasty (10.1% vs 15.7%; odds ratio, 0.60; 95% confidence interval, 0.51-0.71; *P* < .001). Death during the first readmission was also less frequent after THA (0.1% vs 0.7%; *P* = .002). A narrowly defined inpatient principal-diagnosis dislocation indicator did not differ significantly between groups and should not be interpreted as overall dislocation incidence. Index and total 90-day hospital charges were higher with THA.

**Conclusions:**

In this observational claims-based study, THA was associated with a lower observed 90-day inpatient readmission rate than hemiarthroplasty. Residual selection bias may partly explain this association, and these findings do not establish a causal benefit of THA.

**Level of Evidence:**

Level III, Retrospective comparative study.

## Introduction

Femoral neck fractures represent one of the most common and consequential injuries among older adults, with substantial implications for morbidity, mortality, and healthcare utilization. Surgical management typically involves either hemiarthroplasty or total hip arthroplasty (THA), yet the optimal procedure for patients in the younger segment of the elderly population remains a subject of ongoing debate [1,2].

Clinical practice guidelines support THA rather than hemiarthroplasty for carefully selected patients with displaced femoral neck fractures who are independently ambulatory and cognitively able to participate in postoperative recovery; hemiarthroplasty remains appropriate for patients who do not meet these selection criteria. These recommendations are informed by randomized trials and meta-analyses demonstrating modest functional advantages with THA in selected patients, balanced against concerns regarding dislocation, perioperative complications, operative time, and index hospitalization resource use [[3], [4], [5], [6]]. As a result, real-world practice patterns continue to favor hemiarthroplasty, even among patients who may be appropriate candidates for THA [7].

However, much of the existing evidence comparing hemiarthroplasty and THA has focused on short-term in-hospital outcomes or functional scores, with relatively limited attention to postdischarge events and hospital-based healthcare utilization. In particular, 90-day readmissions represent a clinically meaningful outcome that may reflect early complications and recovery trajectory, yet remain underexplored in this population [8]. Although readmissions may influence subsequent hospital utilization, comparisons based on inpatient charges alone cannot establish complete episode-of-care costs or cost-effectiveness because postacute, outpatient, and professional expenditures are not captured [9,10].

Patients aged 65-75 years represent a clinically important subgroup at the intersection of traditional treatment paradigms. Although claims-based comorbidity measures and a coding-derived frailty score can partially characterize recorded illness burden, treatment selection is also strongly influenced by prefracture ambulatory status, independence, cognitive status, frailty, living situation, and surgeon preference. These factors remain incompletely captured in administrative datasets and may confound observational comparisons between THA and hemiarthroplasty [[11], [12], [13]].

The purpose of this study was to compare 90-day inpatient readmission and hospital-based secondary outcomes between hemiarthroplasty and THA among patients aged 65-75 years with femoral neck fractures. We hypothesized that THA use would be associated with a lower observed early inpatient readmission rate after balancing measured demographic and clinical characteristics. We did not seek to establish procedural superiority or comparative cost-effectiveness.

## Material and methods

### Data source

We performed a retrospective cohort study using the 2022 Nationwide Readmissions Database (NRD), a component of the Healthcare Cost and Utilization Project (HCUP) sponsored by the Agency for Healthcare Research and Quality. The NRD is a stratified sample of inpatient discharges from participating U.S. states and is designed to support national estimates; it does not include every U.S. state. The database includes deidentified linkage variables permitting identification of inpatient readmissions within a calendar year. Because the NRD contains deidentified data, this study was exempt from institutional review board review.

### Study population

We identified patients aged 65-75 years with an acute initial-encounter primary diagnosis of femoral neck fracture who underwent hemiarthroplasty or THA during the index hospitalization. Full International Classification of Diseases, Tenth Revision, Clinical Modification (ICD-10-CM) and International Classification of Diseases, Tenth Revision, Procedure Coding System (ICD-10-PCS) code lists are provided in Supplementary Table S1.

The primary cohort included acute femoral neck fracture codes and did not include codes explicitly describing nondisplaced fractures. However, several included intracapsular fracture codes do not explicitly indicate displacement. Accordingly, the main analysis should not be interpreted as restricted exclusively to displaced fractures.

To maximize within-state readmission capture, we restricted the cohort to patients recorded as residents of the same U.S. state in which the index admission occurred. Index admissions were limited to January through September 2022 to permit 90-day follow-up within the same calendar year. Patients were not excluded on the basis of coronavirus disease 2019 (COVID-19) diagnoses during a subsequent hospitalization, thereby avoiding conditioning cohort eligibility on a post-index event.

Readmissions were defined as inpatient admissions occurring after discharge from the index hospitalization and within 90 days. For patients with more than 1 readmission, only the first readmission was retained.

### Propensity score matching

A claims-based Hospital Frailty Risk Score (HFRS) was calculated from the 109 three-character ICD-10 diagnosis categories and published weights described by Gilbert et al [14], using all diagnosis codes recorded during the index hospitalization. Propensity scores for receipt of THA were estimated using logistic regression. The model included HFRS, sex, and the following measured baseline covariates: hypertension, diabetes mellitus, renal disease, congestive heart failure, chronic lung disease, obesity, osteoporosis, liver disease, dementia, Alzheimer disease, Parkinson disease, prior cerebrovascular accident, hemiplegia, peripheral vascular disease, history of myocardial infarction, chronic anemia, smoking, alcohol abuse, drug abuse, mental disorders, sleep apnea, peptic ulcer disease, solid-organ neoplasm, hematologic malignancy, and acquired immunodeficiency syndrome.

Patients undergoing THA were matched 1:1 to patients undergoing hemiarthroplasty using nearest-neighbor matching without replacement, exact matching on age, HFRS risk category (low, intermediate, or high), and mental-disorder diagnosis status, and a caliper of 0.20 standard deviations of the logit of the propensity score. Covariate balance was assessed using standardized mean differences (SMDs). An absolute SMD below 0.10 was prespecified as acceptable. Propensity score overlap before matching is shown in Supplementary Figure S1.

### Outcomes

The primary outcome was any all-cause inpatient readmission within 90 days of index discharge. Secondary outcomes included index length of stay, index hospital charges, death during the first readmission, and first-readmission indicators of prosthetic dislocation, periprosthetic joint infection, periprosthetic fracture, and revision/removal/replacement procedures.

Mechanical outcomes were identified from the principal diagnosis and first recorded procedure of the first inpatient readmission. These narrow indicators do not capture emergency department–only events, outpatient-treated events, or dislocations coded as a secondary diagnosis; they therefore cannot estimate the overall incidence of these complications.

### Hospital charges

Hospital charges were obtained directly from the NRD. Total 90-day hospital charges per index case were calculated as the index hospitalization charge plus the charge from the first 90-day readmission, when present; patients without readmission were assigned zero readmission charges. These data represent hospital charges, not costs, and exclude post-acute, professional, outpatient, and emergency department–only care. Therefore, this analysis is not a formal cost-effectiveness or complete episode-of-care analysis.

### Statistical analysis

Matching and primary outcome analyses were conducted using unweighted discharge-level records. HCUP discharge weights were used only to describe national estimates before matching. Categorical outcomes were compared using chi-square tests and are reported as odds ratios with 95% confidence intervals. Continuous outcomes are reported as mean ± standard deviation and were compared using Welch *t* tests. All tests were two-sided, and *P* < .05 was considered statistically significant.

## Results

### Study cohort and balance

Cohort selection is summarized in Figure 1. The analytic file contained 22,179 January-to-September arthroplasty admissions among patients aged 65-85 years. Restriction to patients aged 65-75 years yielded 8,739 records, and restriction to acute initial-encounter femoral neck fracture diagnoses yielded 8,572 records. After restricting the cohort to state residents, 8,012 eligible index admissions remained, including 5,108 hemiarthroplasties and 2,904 THAs.

**Figure 1:**
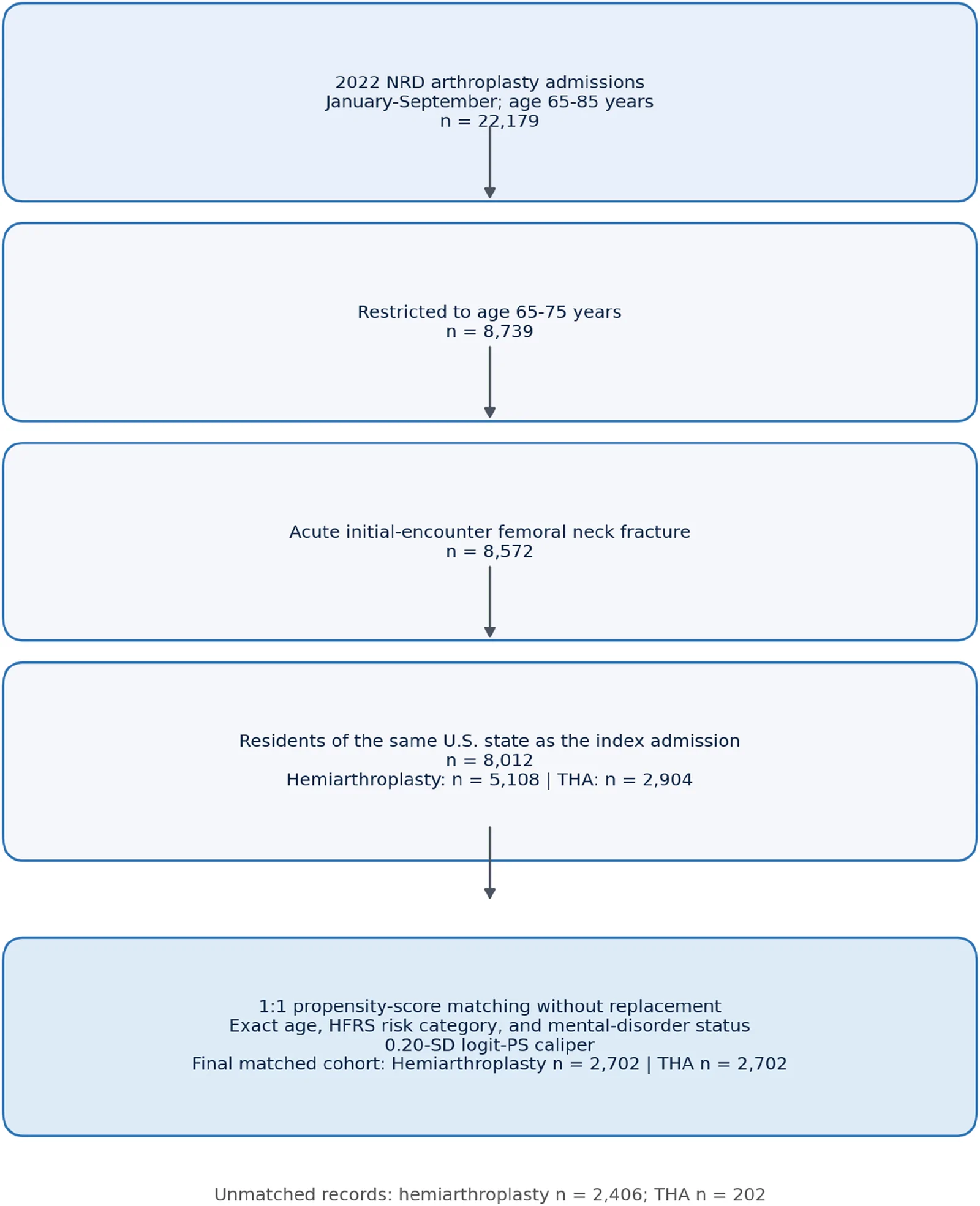
Study flow and derivation of the unweighted propensity-score-matched cohort.

Propensity score matching yielded 2,702 matched pairs. Two hundred two THA records and 2,406 hemiarthroplasty records were not retained in the matched cohort. Baseline characteristics of the matched cohort are presented in Table 1. All modeled covariates achieved acceptable postmatch balance, with absolute SMDs below 0.10 (maximum, 0.040). Full prematch and postmatch balance diagnostics are presented in Supplementary Table S2, and propensity score distributions before matching are shown in Supplementary Figure S1.

**Table 1:** Selected characteristics of the unweighted propensity score–matched cohort.

Characteristic	Hemiarthroplasty (n = 2702)	THA (n = 2702)	Absolute SMD
Age, years	70.46 ± 3.17	70.46 ± 3.17	0.000
Hospital Frailty Risk Score	7.04 ± 3.99	6.93 ± 3.91	0.026
Female sex	63.9%	64.4%	0.011
Mental disorders	48.1%	48.1%	0.000
Dementia	3.9%	3.1%	0.040
Prior cerebrovascular accident	9.5%	8.5%	0.035
Renal disease	12.5%	12.2%	0.009
Congestive heart failure	1.4%	1.4%	0.003
Chronic lung disease	11.3%	10.3%	0.031
Diabetes mellitus	23.0%	22.0%	0.024
Obesity	10.5%	10.8%	0.010
Osteoporosis	13.8%	13.9%	0.001

Full prematch and postmatch balance diagnostics are provided in Supplementary Table S2.

### Index hospitalization outcomes and hospital charges

Index hospitalization outcomes and hospital charges are presented in Table 2. THA was associated with a shorter index length of stay than hemiarthroplasty (5.41 ± 4.68 vs 6.05 ± 5.52 days; *P* < .001). Mean index hospital charges were higher in the THA group ($109,988 ± $84,467 vs $97,246 ± $66,839; *P* < .001).

**Table 2:** Index hospitalization and hospital-charge outcomes in the matched cohort.

Outcome	Hemiarthroplasty	THA	*P*-Value
Index length of stay, days	6.05 ± 5.52	5.41 ± 4.68	<.001
Index hospital charges, USD	97,246 ± 66,839	109,988 ± 84,467	<.001
Total 90-day hospital charges per index case, USD	112,007 ± 94,976	119,021 ± 104,014	.010

Hospital charges are not costs and exclude postacute, professional, outpatient, and emergency department–only care.

Mean total 90-day hospital charges per index case were $119,021 ± $104,014 after THA and $112,007 ± $94,976 after hemiarthroplasty (*P* = .010). Thus, within this matched cohort, total 90-day hospital charges were higher after THA; these charge data do not establish comparative costs or cost-effectiveness.

### Ninety-day readmission outcomes

Ninety-day inpatient readmission outcomes are presented in Table 3. Ninety-day inpatient readmission occurred in 423 hemiarthroplasty patients (15.7%) and 272 THA patients (10.1%). THA use was associated with lower odds of 90-day inpatient readmission than hemiarthroplasty (odds ratio, 0.60; 95% confidence interval, 0.51-0.71; *P* < .001).

**Table 3:** Ninety-day inpatient readmission outcomes in the matched cohort.

Outcome	Hemiarthroplasty (n = 2702)	THA (n = 2702)	*P*-Value
Any 90-day inpatient readmission	423 (15.7%)	272 (10.1%)	<.001
Death during first readmission	19 (0.7%)	4 (0.1%)	.002
Inpatient principal-diagnosis prosthetic dislocation^a^	36 (1.3%)	24 (0.9%)	.119
Periprosthetic joint infection^a^	21 (0.8%)	15 (0.6%)	.316
Periprosthetic fracture^a^	6 (0.2%)	7 (0.3%)	.781
Revision/removal/replacement procedure^a^	71 (2.6%)	45 (1.7%)	.015

a Defined from the principal diagnosis or first recorded procedure of the first inpatient readmission; these narrow indicators do not capture emergency department–only, outpatient, or secondary-diagnosis events and do not estimate overall complication incidence.

Death during the first readmission occurred in 19 hemiarthroplasty patients (0.7%) and 4 THA patients (0.1%; *P* = .002). The inpatient principal-diagnosis indicator for prosthetic dislocation was recorded in 36 hemiarthroplasty patients (1.3%) and 24 THA patients (0.9%; *P* = .119), and should not be interpreted as evidence of a lower overall dislocation risk after THA. Periprosthetic joint infection occurred in 21 hemiarthroplasty patients (0.8%) and 15 THA patients (0.6%; *P* = .316), and periprosthetic fracture occurred in 6 hemiarthroplasty patients (0.2%) and 7 THA patients (0.3%; *P* = .781). A first-readmission revision/removal/replacement procedure was recorded in 71 hemiarthroplasty patients (2.6%) and 45 THA patients (1.7%; *P* = .015).

## Discussion

In this propensity score–matched analysis of patients aged 65-75 years with femoral neck fractures, THA use was associated with a lower observed 90-day inpatient readmission rate than hemiarthroplasty. This association persisted after matching on age, HFRS risk category, mental disorder status, and an expanded range of measured claims-based comorbidities, including dementia, neurologic disease, vascular disease, chronic organ disease, anemia, substance-use diagnoses, and mental disorders. The finding extends prior literature evaluating arthroplasty outcomes and hospital utilization after femoral neck fracture [6,[15], [16], [17]].

A key strength of this study is its focus on a clinically meaningful and frequently debated age group. Patients aged 65-75 years occupy a transitional zone between traditional frail geriatric fracture populations and younger, more active patients. Clinical guidelines and prior comparative studies support consideration of THA in carefully selected patients, particularly those who are cognitively intact and independently ambulatory [6,[18], [19], [20], [21]]. However, these same characteristics influence treatment allocation and cannot be fully measured in an administrative database. Within the available data, propensity score matching provides the closest feasible comparison of measured patient characteristics, but it cannot replace direct measurement of prefracture function.

The lower readmission rate observed after THA should therefore not be interpreted as evidence that THA itself caused a superior postoperative course. Incorporating HFRS and coded neurologic/cognitive conditions improves adjustment for recorded illness burden, but does not measure prefracture ambulatory status, independence in activities of daily living, use of ambulatory aids, living situation, detailed cognition, surgeon preference, or patient goals. The HFRS is a claims-based risk surrogate, not a clinical frailty phenotype. Residual confounding by indication is therefore likely, and patients selected for THA may have been healthier, more active, and functionally better suited for THA than those receiving hemiarthroplasty [13].

The present findings should also be interpreted in the context of high-quality evidence documenting an important dislocation trade-off with THA. The Hip Fracture Evaluation with Alternatives of Total Hip Arthroplasty vs Hemiarthroplasty (HEALTH) trial found higher dislocation risk with THA among independently ambulatory patients [5]. Edelstein et al [17] similarly found higher dislocation risk after THA in an instrumental-variable Medicare analysis, and a systematic review by Lewis et al [6]. reported a greater early dislocation risk with THA. In contrast, the present inpatient principal-diagnosis indicator had a numerically lower, but nonsignificant, rate after THA (0.9% vs 1.3%). This discordant direction should not be regarded as a clinical incidence estimate or a reassuring safety signal: the NRD misses emergency department–only and outpatient events, events managed without admission, and events not coded as the principal diagnosis. The finding instead underscores severe under-ascertainment of this outcome and the possibility of residual treatment-selection bias [[22], [23], [24]].

Mortality during the first readmission was lower in the THA group, although the absolute number of events was small. This result should not be interpreted as all-cause 90-day mortality because the NRD captures only deaths occurring during an inpatient readmission. The observed difference may reflect residual differences in frailty, functional reserve, and patient selection rather than a survival effect of the procedure itself [25,26].

The hospital-charge analysis does not support a conclusion that THA provides superior economic value. Although THA was associated with higher index and total 90-day hospital charges in the matched cohort, hospital charges are not equivalent to costs and exclude skilled-nursing care, inpatient rehabilitation, professional fees, outpatient services, and emergency department care. Therefore, these findings should be interpreted as hospital-based utilization data rather than a complete episode-of-care cost or cost-effectiveness analysis [27,28].

This study has several limitations. HFRS was incorporated as a claims-based frailty-risk surrogate; however, it was calculated from diagnoses recorded during the index hospitalization, and the NRD lacks present-on-admission indicators and the historical look-back data needed to fully characterize prefracture frailty. Claims data also do not measure prefracture ambulatory status, use of ambulatory aids, functional independence, living arrangement, detailed cognitive status, surgeon preference, or patient goals. The NRD captures inpatient readmissions only within participating states and within the same calendar year; out-of-state, outpatient, emergency department–only, and later events may be missed. Patient-reported outcomes, functional recovery, radiographic findings, surgical approach, implant details, and long-term revision outcomes were unavailable. In addition, hospital charges are not costs and exclude substantial components of postfracture care.

Despite these limitations, this study provides a large contemporary comparison of hospital-based outcomes in a narrowly defined age group, uses expanded measured-confounder adjustment including a claims-based frailty-risk score, explicitly reports matching balance and propensity score overlap, and carefully limits interpretation of readmission-specific mechanical indicators.

## Conclusions

Among patients aged 65-75 years with femoral neck fracture who were balanced on measured claims-based characteristics, THA use was associated with a lower observed 90-day inpatient readmission rate than hemiarthroplasty. These findings should be interpreted in the context of patient selection, as patients undergoing THA may have been healthier and more functionally active. Treatment selection should not be based on age alone; functional status, pre-injury activity level, cognitive and mental capacity, and overall health remain important considerations when selecting between THA and hemiarthroplasty. This observational association should not be interpreted as proof that THA is causally superior. Prospective studies incorporating prefracture function, cognition, frailty, patient-reported outcomes, and complete episode-of-care costs are required to refine procedure selection.

## Ethics approval

Ethics approval and consent to participate: The NRD contains deidentified data. This study was exempt from institutional review board review.

## Data availability

The NRD is available from HCUP subject to a data-use agreement. Full code lists and balance diagnostics are provided in the Supplementary Material.

## CRediT authorship contribution statement

**David Maman:** Writing – original draft, Formal analysis, Data curation, Conceptualization. **Yaniv Steinfeld:** Writing – review & editing, Supervision, Conceptualization. **Yaron Berkovich:** Writing – review & editing, Supervision, Methodology.

## Conflicts of interest

The authors declare no potential conflicts of interest.

For full disclosure statements refer to https://doi.org/10.1016/j.artd.2026.102168.

## Figures and Tables

**Supplementary Table S1:** ICD-10-CM and ICD-10-PCS code lists.

Category	Codes
Acute femoral neck fracture diagnosis	S72.001A, S72.002A, S72.011A, S72.012A, S72.031A, S72.032A, S72.041A, S72.042A, S72.051A, S72.052A, S72.091A, S72.092A
Hemiarthroplasty procedures	0SRR019, 0SRR01A, 0SRR01Z, 0SRR039, 0SRR03A, 0SRR03Z, 0SRR0J9, 0SRR0JA, 0SRR0JZ, 0SRS019, 0SRS01A, 0SRS01Z, 0SRS039, 0SRS03A, 0SRS03Z, 0SRS07Z, 0SRS0J9, 0SRS0JA, 0SRS0JZ, 0SRS0KZ
Total hip arthroplasty procedures	0SR9019, 0SR901A, 0SR901Z, 0SR9029, 0SR902A, 0SR902Z, 0SR9039, 0SR903A, 0SR903Z, 0SR9049, 0SR904A, 0SR904Z, 0SR9069, 0SR906A, 0SR906Z, 0SR907Z, 0SR90EZ, 0SR90J9, 0SR90JA, 0SR90JZ, 0SRB019, 0SRB01A, 0SRB01Z, 0SRB029, 0SRB02A, 0SRB02Z, 0SRB039, 0SRB03A, 0SRB03Z, 0SRB049, 0SRB04A, 0SRB04Z, 0SRB069, 0SRB06A, 0SRB06Z, 0SRB0J9, 0SRB0JA, 0SRB0JZ, 0SRB0KZ

The primary cohort did not include codes explicitly identifying a nondisplaced femoral neck fracture. Some included intracapsular fracture codes do not specify displacement; the main cohort is therefore not described as exclusively displaced.

**Supplementary Table S2:** Full covariate balance after matching.

Covariate	Pre-match SMD	Post-match SMD
Age	−0.307	0.000
Female sex	0.029	0.011
Hypertension	0.000	−0.001
Diabetes mellitus	−0.151	−0.024
Renal disease	−0.110	−0.009
Congestive heart failure	−0.117	−0.003
Chronic lung disease	−0.246	−0.031
Obesity	0.045	0.010
Osteoporosis	−0.001	0.001
Liver disease	−0.071	−0.024
Dementia	−0.240	−0.040
Alzheimer disease	−0.178	−0.003
Parkinson disease	−0.160	−0.007
Prior cerebrovascular accident	−0.194	−0.035
Hemiplegia	−0.028	−0.027
Peripheral vascular disease	−0.072	0.012
History of myocardial infarction	−0.034	0.013
Chronic anemia	−0.063	0.002
Smoking	−0.022	−0.012
Drug abuse	0.000	0.000
Alcohol abuse	−0.106	−0.018
Mental disorders	−0.304	0.000
Sleep apnea	0.006	0.005
Peptic ulcer disease	−0.014	0.006
Solid-organ neoplasm	−0.102	−0.012
Hematologic malignancy	−0.016	−0.018
AIDS	0.000	0.000
Hospital Frailty Risk Score	−0.447	−0.026

All modeled covariates, including HFRS, had post-match absolute standardized mean differences below 0.10 (maximum, 0.040).

**Supplementary Table S3:** Hospital Frailty Risk Score ICD-10 categories and weights.

ICD-10	Weight	ICD-10	Weight	ICD-10	Weight
F00	7.1	G81	4.4	G30	4.0
I69	3.7	R29	3.6	N39	3.2
F05	3.2	W19	3.2	S00	3.2
R31	3.0	B96	2.9	R41	2.7
R26	2.6	I67	2.6	R56	2.6
R40	2.5	T83	2.4	S06	2.4
S42	2.3	E87	2.3	M25	2.3
E86	2.3	R54	2.2	Z50	2.1
F03	2.1	W18	2.1	Z75	2.0
F01	2.0	S80	2.0	L03	2.0
H54	1.9	E53	1.9	Z60	1.8
G20	1.8	R55	1.8	S22	1.8
K59	1.8	N17	1.8	L89	1.7
Z22	1.7	B95	1.7	N19	1.6
A41	1.6	L97	1.6	R44	1.6
K26	1.6	I95	1.6	Z87	1.5
J96	1.5	X59	1.5	M19	1.5
G40	1.5	M81	1.4	S72	1.4
S32	1.4	E16	1.4	R94	1.4
N18	1.4	R33	1.3	R69	1.3
N28	1.3	R32	1.2	G31	1.2
Y95	1.2	S09	1.2	R45	1.2
G45	1.2	S01	1.1	A04	1.1
A09	1.1	J18	1.1	Z74	1.1
M79	1.1	W06	1.1	J69	1.0
R47	1.0	E55	1.0	Z93	1.0
R02	1.0	R63	1.0	H91	0.9
W10	0.9	W01	0.9	E05	0.9
M41	0.9	R13	0.8	Z99	0.8
U80	0.8	M80	0.8	K92	0.8
I63	0.8	J22	0.7	N20	0.7
F10	0.7	Y84	0.7	R00	0.7
Z73	0.6	R79	0.6	Z91	0.5
S51	0.5	F32	0.5	M48	0.5
E83	0.4	M15	0.4	D64	0.4
L08	0.4	R11	0.3	K52	0.3
R50	0.1				

Weights are from Gilbert et al. (Lancet. 2018;391:1775-1782). HFRS was calculated from diagnosis codes recorded during the index hospitalization.

**Supplementary Figure S1:**
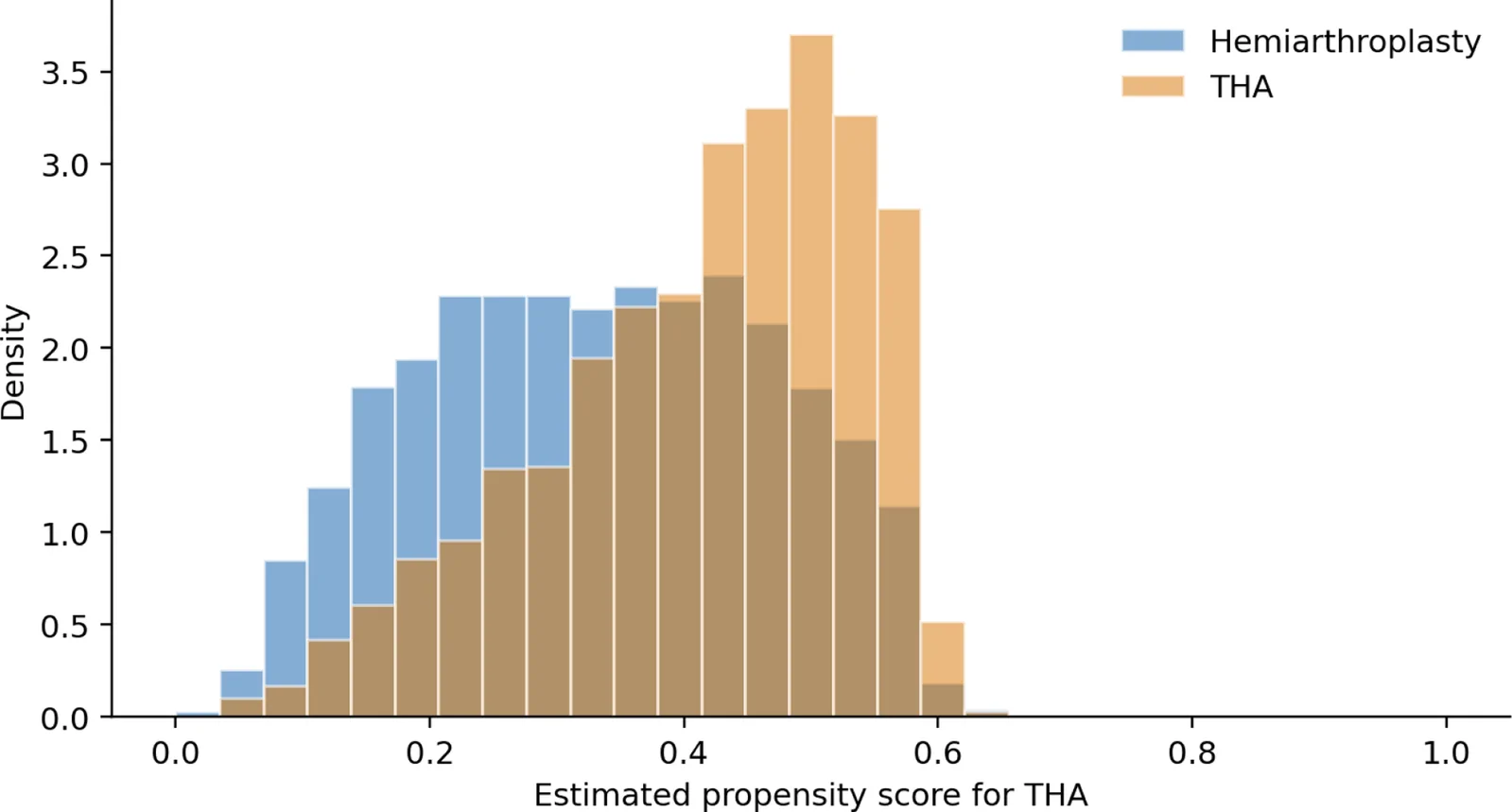
Propensity-score distribution before matching.The plot shows the estimated propensity score for THA in the eligible cohort before matching. Matching was performed without replacement within exact strata of age, HFRS risk category, and mental-disorder diagnosis status, using a 0.20-SD logit propensity-score caliper.

## Appendix A Supplementary data

Conflict of Interest Statement for Berkovich

Conflict of Interest Statement for Maman

Conflict of Interest Statement for Steinfeld
