# Supplementary material for: Total Hip Arthroplasty Is Associated With Lower 90-Day Inpatient Readmission Than Hemiarthroplasty for Femoral Neck Fracture in Patients Aged 65-75 Years: A Propensity Score-Matched Nationwide Readmissions Database Study
Source: Arthroplast Today. 2026 Oct 6;41:102168. doi: 10.1016/j.artd.2026.102168 (PMC13673019; doi:10.1016/j.artd.2026.102168)
Supplement: Supplementary file 3 [file mmc3.docx]

# CONFLICT OF INTEREST STATEMENT

American Association of Hip and Knee Surgeons
(Adopted from the American Academy of Orthopaedic Surgeons disclosure statement)

Manuscript Title:
Total Hip Arthroplasty Is Associated With Lower Readmission Burden and Improved Early Value Compared With Hemiarthroplasty in Patients Aged 65-75 Years With Femoral Neck Fractures

1. Royalties from a company or supplier — None.

2. Speakers bureau/paid presentations for a company or supplier — None.

3A. Paid employee for a company or supplier — None.

3B. Paid consultant for a company or supplier — None.

3C. Unpaid consultant for a company or supplier — None.

4. Stock or stock options in a company or supplier — None.

5. Research support from a company or supplier as a Principal Investigator — None.

6. Other financial or material support from a company or supplier — None.

7. Royalties, financial or material support from publishers — None.

8. Medical/Orthopaedic publications editorial/governing board — None.

9. Board member/committee appointments for a society — None.

Author Name (Print or Type): Yaniv Steinfeld, MD

Author Signature: _

Date: 28.12.2025
